# Building an implementation framework for directly observed feedback by attending physicians

**DOI:** 10.1371/journal.pone.0342550

**Published:** 2026-02-09

**Authors:** Andrew Vincent Raikhel, Helene Starks, Gabrielle Berger, Jeffrey Redinger

**Affiliations:** 1 Division of General Internal Medicine, University of Washington, Seattle, Washington, United States of America; 2 Department of Bioethics and Humanities, University of Washington, Seattle, Washington, United States of America; Iuliu Hațieganu University of Medicine and Pharmacy: Universitatea de Medicina si Farmacie Iuliu Hatieganu, ROMANIA

## Abstract

**Background:**

Effective formative feedback from attending physicians to residents is critical for competency-based medical education. Feedback curricula commonly focus on simulated feedback delivery while actual verbal feedback delivery is unobserved by anyone other than the individuals involved. External observation of feedback has received limited attention as a novel method of improving feedback quality. Despite this, there is no research describing attitudes towards directly observed feedback.

**Methods:**

We developed two surveys, one for Internal Medicine residents (IMRs) and one for hospitalists, who specialize in comprehensive care of hospitalized patients, at the University of Washington in Seattle, Washington in 2023. Survey validity evidence was gathered prior to disseminating surveys via a census sampling approach by group email listservs. Quantitative questions were analyzed by dichotomizing Likert responses as neutral/disagree vs. agree. Free text comments were qualitatively analyzed via a general inductive approach until theme sufficiency was reached. Survey development and analysis was conducted using a lens of social cognitive theory.

**Results:**

The response rate was 71% (130/184) and 57% (74/129) for IMRs and hospitalists respectively. Most residents and hospitalists reported feeling comfortable with having a feedback exchange observed (105/129, 81%; 46/72, 64% respectively). Hospitalist and IMR concerns about the implementation of directly observed feedback were categorized into three themes: concerns about the relationship with the faculty observer, negative impact on learning environment, and altered feedback quality. Hospitalist and IMR suggestions for parameters to mitigate the challenges of observed feedback were categorized into three themes: the feedback observer’s relational boundaries, empower participant agency, and preserve feedback integrity.

**Conclusion:**

The thematic concerns expressed by both cohorts relate to social monitoring, either of a projected self-image or to the educational safety of a learner. These themes highlight the fundamental importance of psychological safety in developing a program of directly observed feedback for attending physicians and residents.

## Introduction

Effective formative feedback from attending physicians to residents is a critical component of competency-based medical education. Best practices of verbal feedback, including specificity and the development of an educational alliance, are established in the feedback lexicon while feedback training has become a common focus of faculty development [1,2]. Despite this, a persistent gap exists across medical specialties between faculty and learner perceptions of feedback quality: attendings often rate their feedback delivery skills favorably while residents consistently note opportunities for improvement [3–8]. Given its dependence on complex social interactions between participants who may be subject to inattention and bias, assessing the quality of feedback delivery and its role in reception of feedback is challenging.

What, then, is the best approach for programmatic improvement of verbal feedback delivery? Feedback improvement curricula commonly focus on simulations of feedback delivery or reflections on feedback that was previously delivered, yet verbal feedback delivery is almost never witnessed by anyone other than the two individuals involved [9]. This makes verbal feedback an outlier within a deliberate practice model, in which direct observation is an expected part of skill development to minimize internal bias from self-assessment alone [10]. External observation of feedback delivery has received recent attention as a novel method of improving verbal feedback quality. Halman et al. developed a rating scale with extensive validity evidence to assess faculty verbal feedback quality [11], while Cardella et al. investigated videorecorded sessions as part of a resident feedback improvement pilot [12]. Despite this, there is no research describing resident or faculty attitudes towards directly observed feedback and no published frameworks to guide implementation.

The contemporary model of high-quality feedback in graduate medical education is that of a bidirectional alliance between attending and resident, in which both parties share a mutual understanding of the resident’s goals and work together to develop strategies to reach those goals [2]. This model grew out of the recognition that feedback encounters occur as social interactions, in which feedback efficacy is not merely dependent on unidirectional transmittal of information but also on sociocultural and cognitive circumstances that affect the perceived authentic engagement of an attending physician.

Because the addition of a third-party observer may impact these complex social dynamics, social cognitive theory (SCT) offers a valuable lens to analyze its effects. SCT, described by Bandura as an extension of social learning theory, frames individuals as active agents who regulate their own learning through social support [13]. The constructs of self-efficacy (belief in one’s ability to succeed in a given skill) and self-regulation are central to SCT, forming the principle that learners can be taught to regulate themselves towards competency through planning, performance, and reflection [14]. SCT also posits that behaviors are influenced by reciprocal determinism: learner actions interact continuously with personal factors and environment as a way to regulate future behavior. Applied to an externally observed feedback environment, SCT predicts a range of possible effects, for instance by amplifying or dampening self-efficacy, outcome expectations, or social reinforcement surrounding feedback delivery or reception.

Given these complex dynamics, we sought to develop an implementation framework using a lens of SCT for a program of directly observed feedback in which a faculty observer watches attending-resident feedback in real-time to enhance future feedback delivery. Rather than framing uptake as a matter of logistics or compliance, we approached implementation as a relational challenge: how might such a program be designed to support social learning dynamics for all participants? To explore this question, we surveyed hospitalists, i.e., physicians whose primary professional focus is the general medical care of hospitalized patients, and internal medicine residents (IMRs) at the University of Washington in Seattle, Washington to assess perceived barriers and optimal parameters for implementation of a program of directly observed feedback.

## Methods

### Survey development and implementation

We conducted a cross-sectional mixed methods study utilizing an anonymous web-based survey of faculty hospitalists and IMRs. The study was conducted at the University of Washington in Seattle, Washington from April to October in 2023 using a census sampling approach. There were no local or national regulations governing methods of feedback delivery during our study, though verbal feedback delivery is an expected responsibility of hospitalist physicians working with IMRs in our local setting. We developed two parallel surveys, one for IMRs and one for hospitalists that collected both quantitative and qualitative data. The surveys aimed to characterize attitudes, barriers, and design preferences for a program of directly observed feedback in which a faculty observer watches another attending physician delivering feedback to a resident in real-time. Survey development was informed by a literature review of current verbal and observed feedback practices. Best practices for survey design were adhered to throughout the survey development process [15]. Surveys were trialed with representative members of both cohorts for content validity, response process validity evidence including clarity of instructions, useability, and timing through a process of cognitive testing. Surveys were iteratively revised throughout the development process. Survey instructions included a definition of formative feedback. Given previous literature describing observation via either in-person assessment or recorded feedback, the surveys included Likert-style questions regarding comfort level in direct observation or videorecording of feedback. Open-ended questions queried IMR and hospitalist perspectives and concerns about being observed by an attending physician feedback observer during reception or delivery of formative feedback. The surveys also included open-ended questions asking how a program of directly observed feedback should be implemented to mitigate any concerns.

Surveys were emailed to the IMR and hospitalist cohorts via group listservs in April and May of 2023 which included all 184 IMRs at a single Internal Medicine residency program. The faculty survey was emailed to all 129 hospitalists who supervise surveyed IMRs on general medicine inpatient rotations at an academic center, a county safety net hospital, and a Veterans Affairs hospital in September and October of 2023. A consent agreement for survey participants was included at the beginning of the survey. Both cohorts were incentivized to participate via gift cards distributed to a limited number of randomly selected survey participants, with a $50 gift card randomly awarded to four residents who completed the survey and a $100 gift card randomly awarded to one hospitalist who completed the survey. The survey was deemed exempt by the University of Washington Institutional Review Board. Informed consent was obtained through an information sheet presented before the survey. No written or verbal documentation of consent was required under our institution’s policy.

### Data analysis

Quantitative questions were analyzed by dichotomizing the Likert responses as neutral/disagree vs. agree. Analysis was performed using Microsoft Excel®. Free text comments were qualitatively analyzed via a general inductive approach with iterative review by two coders (AVR and JR) whose analysis was informed by the principles of SCT, which conceptualizes learning as a socially situated and self-regulated process in which individuals adapt their behavior based on observation, perceived outcomes, social reinforcement, and self-efficacy. Initial coding was inductive and conducted independently by both coders with bimonthly memo-supported discussions over two months to resolve differences and update the codebook. Inductive codes were created in the process of survey analysis; codes were continually added, refined, and applied to the data in an iterative manner. Differences in coding were tracked in a shared audit log. Consensus in coding was reached through structured and deliberate discussion between the two coders with frequency of comments tabulated by code. After completion of coding, raters independently clustered codes into higher-order categories and met to integrate these into emerging themes. Theme development proceeded until theme sufficiency was reached. We specifically attended to themes reflecting relational and contextual dynamics of the feedback encounter, in keeping with our interest in understanding how feedback behaviors are shaped within a social cognitive context.

## Results

Of the 184 IMRs asked to complete the survey we received 130 responses for a response rate of 71%. One IMR response was excluded due to an incorrect attention check question. Of the 129 academic hospitalists surveyed we received 74 responses for a response rate of 57%. Two hospitalist responses were excluded due to reporting working zero weeks per year with IMRs. Of 72 surveyed hospitalists, 58% were women (43/72). Time since completion of residency training for hospitalists spanned from 1–2 years (18% of respondents), 3–5 years (22%), 6–10 years (29%), 11–20 years (24%) and >21 years (4%). Annual involvement supervising residents during inpatient clinical care ranged from 1–2 weeks (8%), 3–5 weeks (22%), 6–10 weeks (36%), 11–20 weeks (18%), and >21 weeks (13%). Overall, 57% of resident respondents identified as women (73/129) and 2% as nonbinary (3/129).Resident respondents included 34 first-year residents (36%), 40 second-year residents (26%), 40 third-year residents (31%), and 9 fourth-year residents (7%).

**Fig 1:**
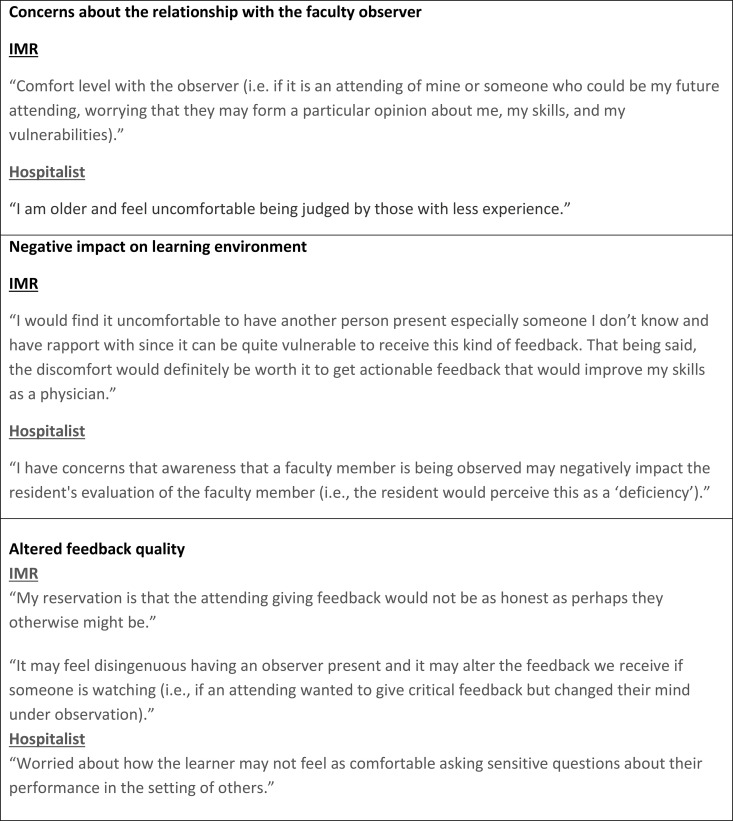
Themes with selected quotes from internal medicine resident (IMR) and hospitalists on their concerns about verbal feedback being observed by a faculty feedback observer.

**Fig 2:**
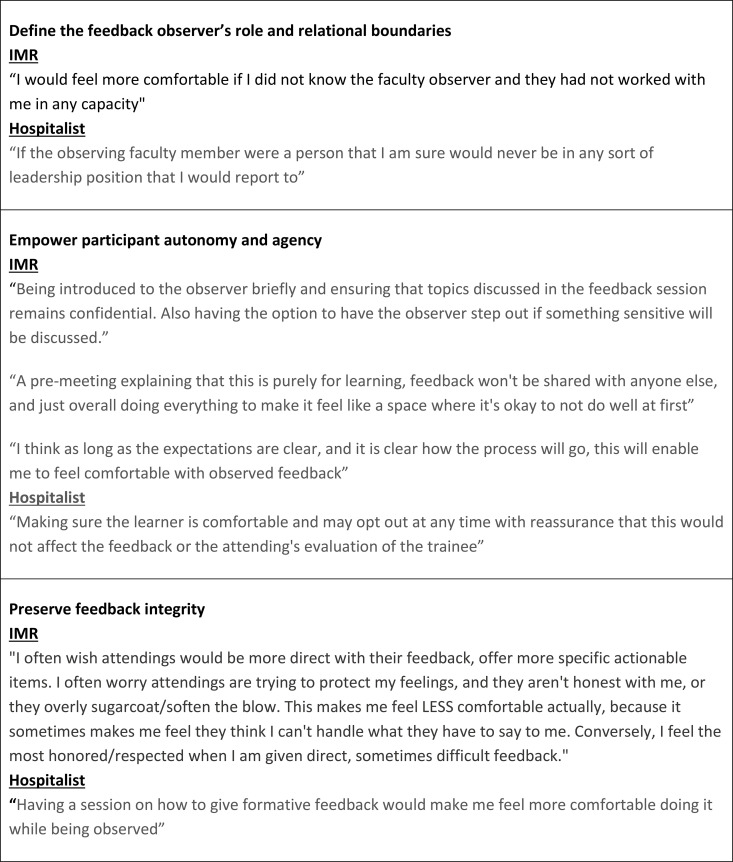
Selected quotes from internal medicine resident (IMR) and hospitalists on strategies that would mitigate concerns about verbal feedback being observed by a faculty feedback observer.

Most residents and hospitalists reported feeling comfortable with having a feedback exchange observed (105/129, 81%; 46/72, 64% respectively). Hospitalist and IMR concerns about the implementation of direct observation of feedback delivery were categorized into three themes: concerns about the relationship with the faculty observer, negative impact on learning environment, and altered feedback quality. Hospitalist and IMR suggestions for parameters to mitigate the challenges of directly observed feedback were categorized into three themes: define the feedback observer’s role and relational boundaries, empower participant autonomy and agency, and preserve feedback integrity. Selected quotes from the survey participants can be seen in Figs 1 and 2.

### Concerns about directly observed feedback

#### Concerns about the relationship with the faculty observer.

Hospitalists and IMRs expressed concerns that a feedback observer with an administrative or supervisory role could negatively impact the workplace environment or evaluations in the future. Some IMRs noted discomfort about future supervision by an attending who observed their feedback, after having discussed their vulnerabilities during the feedback session. Residents were concerned that if feedback observers were involved in residency leadership, the experience could unintentionally impact how the IMR is perceived by the residency program more broadly. Hospitalists expressed worry that if feedback observers were involved in hospital leadership, the observations could be more akin to a performance review. Some hospitalists noted that they would have reservations being observed by colleagues who had less clinical teaching experience.

#### Negative impact on learning environment.

Both IMR and hospitalists reported concerns regarding IMRs feeling more vulnerable receiving feedback in the setting of a third-party observer, which could impact their growth mindset. Hospitalists reported concerns that their faculty evaluations from learners may be negatively impacted if learners assumed that participation in the program stemmed from a need for faculty remediation.

#### Altered feedback quality.

Respondents cited alteration of feedback content due to a third party’s presence as a possible challenge. Residents noted that observed feedback may be more complimentary and less specific to avoid making the learner uncomfortable. Hospitalists were concerned that IMRs may be less willing to engage in a conversation about the content of the feedback in the setting of an observation.

### Mitigating barriers to a program of directly observed feedback

Hospitalist and IMR suggestions for implementing a program of directly observed feedback were categorized into three themes: define the feedback observer’s role and relational boundaries, empower participant autonomy and agency, and preserve feedback integrity.

#### Define the feedback observer’s role and relational boundaries.

Hospitalists and IMRs thought that the feedback observer should have a nonevaluative relationship with the learner and should not have any supervisory role with the learner or faculty being observed. Some hospitalists suggest that the ideal feedback observer has a near-peer relationship with the faculty being observed as opposed to being an identified ‘expert’ or senior faculty member to reduce risk of intimidation impacting feedback. To support residents, it was suggested that the feedback observer should introduce themselves to the learner prior to the feedback session, giving the learner an opportunity to ask questions and understand the session’s goals. Both cohorts suggested that the feedback observer should be nonobtrusive to the feedback conversation.

#### Empower participant autonomy and agency.

Hospitalists and IMRs said that the specific feedback delivered to the learner should remain confidential. Both cohorts said that assurances that the observation’s purpose is to develop the attending’s skill in delivering feedback should be made clear to the resident and attending. Hospitalists also advocated for strong messaging around this program’s availability to all faculty as a voluntary learning opportunity to improve their teaching skills, rather than participation being required for remediation.

Additionally, both cohorts cited the importance of empowering the learner to dismiss the faculty observer at any time if the learner becomes uncomfortable with the triadic dynamic during a directly observed feedback session.

#### Preserve feedback integrity.

Both IMRs and hospitalists said that the specifics of the feedback delivered, as much as possible, should not be impacted by a third-party observer. Both cohorts suggested that learners requiring feedback on professional behavior, or learners who require structural remediation to their educational plans should not be included in a program of direct observation. Instead, the ideal feedback content for a program of directly observed feedback should be focused on actionable skill building. Several hospitalists suggested that a program of direct observation should be paired with more traditional didactics that teach the best practices of feedback delivery.

### Modelling a relationship between teaching and learner phycological safety, and the impact on directly observed feedback

**Fig 3:**
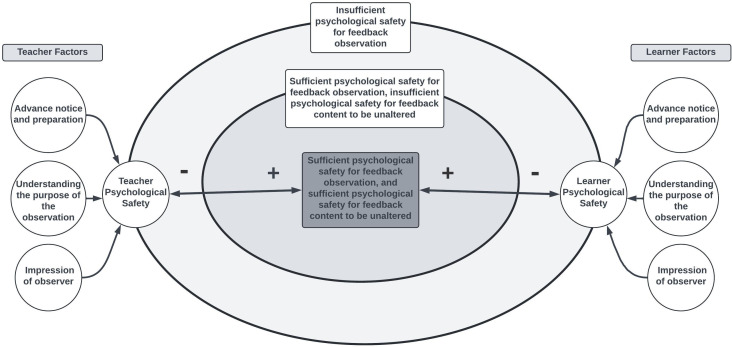
A proposed model for factors that impact the psychological safety of teacher and learners in the setting of directly observed feedback delivery. In this model, these factors impact teacher and learner independently and influence the degree of psychological safety. The degree of psychological safety sets the stage for a feedback observation. If there is insufficient psychological safety, it is unlikely that a feedback observation will be a productive tool for improvement and may be detrimental to either the learner or teacher. If there is a high degree of psychological safety, the feedback observation can likely occur and there will be minimal impact on the feedback content as a result of the observation. If the psychological safety is somewhere in the middle, a feedback observation may occur, but the feedback content may be more heavily influenced by the observation.

Fig 3 models the relationship of learner and teacher inputs of psychological safety and how these can coalesce to influence the environment of observed feedback.

## Discussion

Most hospitalists and IMRs reported comfort with a novel feedback observation program while identifying pitfalls and mitigating strategies to strengthen implementation. The thematic concerns expressed by both surveyed cohorts broadly relate to social monitoring, either of a projected self-image to colleagues and supervisors or to the educational safety of a learner. A lens of psychological safety as situated within a social cognitive learning environment, then, is a helpful construct to understand our results. Psychological safety, i.e., a feeling of comfort to take risks without fear of damage to self-image, status, or career, empowers interpersonal risk-taking and reflects an underlying culture of trust [15]. In medical education, supervising physicians are thought to exert immense influence over the psychological safety of a learning environment [16,17]. It is no surprise, then, that both IMRs and hospitalists focused on the potential social complexities of an observation, with themes highlighting the potential risks of an inauthentic feedback exchange or unintended effects on summative evaluation by the third-party observer. Interestingly, attending physicians also voiced concerns regarding their own psychological safety while being observed, indicating that a need to mitigate these concerns applies to both parties involved in the feedback exchange.

Despite these documented concerns, a majority of IMRs and hospitalists reported comfort with a novel program of directly observed feedback and, importantly, provided a roadmap to navigate potential pitfalls. The themes for implementation identified in our study are again best understood through a psychological safety construct [18]. The cohorts’ shared strategies for mitigating barriers to observed feedback, including advanced notice, transparent expectations, curated faculty dyad pairings, and minimal power distance between faculty, are reminiscent of strategies used to maximize psychological safety in medical education learning environments [15]. These strategies can expand the impact of feedback conversations beyond the usual goal of discussion aimed at improving learner skills or knowledge, as the learner has the opportunity to see their teacher modelling growth through their own engagement in feedback observation. Additionally, the feedback observer has an opportunity to see models of feedback delivery from the teacher that may impact their feedback practice. These relational interactions have the potential to increase the impact of feedback exponentially via modeling and social reciprocal determinism engendered by having feedback observed by a third-party.

These organizing principles can be synthesized together into a foundation to support the development of a directly observed feedback program. Just as the psychological safety of a learning environment can either jeopardize or amplify educational objectives, our themes support a model for how safety within directly observed feedback may alter fundamental aspects of the feedback content or the relationship between the resident and faculty. If insufficient care is employed in the implementation of directly observed feedback, the inclusion of a third party for observation may subvert the learning climate and make an honest feedback exchange more challenging, or possibly deleterious to the learner. This is vital to consider given the increased exploration of direct observation as a novel tool for improving feedback skills. As with many social phenomena, our model does not suggest that the success of directly observed feedback to be a binary choice. Rather, optimizing the learning environment to support the successful implementation of directly observed feedback exists on a continuum, impacted by independent learner and teacher factors. While we have extrapolated barriers and considerations for implementation that can be implemented generally, local learning environments and unique social dynamics may increase the significance and weight of some of the elements in our model. This may necessitate local adaptations and tailoring of the model to meet the needs of some learning environments.

Not all challenges in implementing a directly observed feedback program could be anticipated by the surveyed group as the program was posed as a hypothetical. However, our findings provide a strong foundation for further work to be undertaken. Our results are limited in generalizability given that we surveyed a single academic program. However, the proposed model aligns with the limited work in medical education that has been completed to this point in the field of directly observed feedback [10,11]. Additionally, our model has face validity when applied to examples of directly observed feedback that exist in fields outside of medical education [18–20]. To test the validity of these findings in practice, they should be implemented in a program aimed at improving faculty feedback through direct observation. After this foundational work has been completed and analyzed, the challenges and opportunities will be better situated for implementation in a variety of learning environments. Surprisingly, no individual comments or identified themes were related to the time investment of such a project, despite the need for two faculty members to be present for each observed feedback session. This could relate to the overriding power of psychological safety within our hypothetical feedback environment but may also be an indicator of how highly regarded feedback improvement and delivery are within our study population. Nonetheless, psychological safety is likely to be an important component of directly observed feedback in other medical education contexts and has practical utility in the design of future programs of externally observed feedback.

Our study has several limitations. It was conducted at a single academic center which limits the generalizability of our findings, though this is mitigated by a robust response rate and relatively large residency program size. Selection bias was also more likely to have occurred within the surveyed attending cohort given the decreased response rate compared to resident physicians, in particular due to attending nonresponse bias. Finally, social desirability bias may have skewed results within either cohort.

Improving the quality of feedback delivery has proven to be a refractory and challenging goal for medical education. Given the established limitations of self-assessment, directly observed feedback may be a powerful new tool in supporting this vital component of teaching clinical medicine. Considering how important high-quality feedback is to professional development in medicine, investigating these questions are worthy next steps to support the competency-based growth of resident physicians.
